# Seeking sufficient and appropriate care during the first year after spinal cord injury: a qualitative study

**DOI:** 10.1038/s41393-024-00974-x

**Published:** 2024-03-16

**Authors:** Anne M. Bryden, Brian Gran

**Affiliations:** 1 MetroHealth Center for Rehabilitation Research, MetroHealth System, Cleveland, OH USA; 2 Department of Physical Medicine and Rehabilitation, Case Western Reserve University School of Medicine, Cleveland, OH USA; 3 Department of Sociology, Case Western Reserve University College of Arts and Sciences, Cleveland, OH USA; 4 School of Law, Case Western Reserve University, Cleveland, OH USA

**Keywords:** Health sciences, Quality of life, Outcomes research

## Abstract

**Study design:**

Longitudinal qualitative study, based on a constructivist grounded theory and transformative approach.

**Objectives:**

This study investigated experiences of individuals with spinal cord injury (SCI) while navigating rehabilitation, resources for recovery, and community reintegration during the first year after injury.

**Setting:**

An acute inpatient rehabilitation facility in the Midwest United States.

**Methods:**

In-depth, semi-structured interviews were conducted with 20 individuals with newly-acquired SCI. Interviews were conducted approximately every other month for one year, beginning at acute inpatient rehabilitation. Data were analyzed and interpreted using a constructivist grounded theory approach and transformative paradigm, which examines power and social structures within and across institutions and gives voice to people at risk for marginalization.

**Results:**

Participants experienced variable post-injury trajectories, with an average of four transitions within and across healthcare institutions in the first three months. Half of the cohort was discharged to a skilled nursing facility (SNF). Emergent themes included discharge (un)readiness; length of stay uncertainty and insurance impacts; challenges choosing a SNF including time-sensitive decisions; and early cessation of therapy in the SNF. Participants experienced resource navigation challenges such as communication/information access barriers and contending with many concerns at once.

**Conclusions:**

The experiences of this cohort reveal significant challenges to attaining sufficient and appropriate rehabilitation. Acute inpatient rehabilitation is a critical aspect of recovery, but does not ensure sufficient intervention for maximization of functional skills and community reintegration. Innovative rehabilitation models need to be developed for positive impacts on successful transition to independent living in the community.

## Introduction

Spinal cord injury (SCI) is a complex condition that challenges an individual’s societal reintegration. Little is known about the lived experiences of people with SCI and their support persons as they seek knowledge about their injury, interventions, and prognosis for recovery.

### Challenges to sufficient and appropriate care

Attaining sufficient and appropriate care in the United States after SCI is increasingly difficult. Scholars report that significant changes in healthcare since the early 1990s have resulted in shorter periods of inpatient rehabilitation following SCI [1–4]. Coupled with shorter periods of acute care management prior to entering inpatient rehabilitation, people with SCI are discharged into the community earlier, and perhaps more acutely ill than ever [3, 5]. As a result, increases in rehospitalizations due to complications from secondary conditions such as urinary tract infections, pressure injuries, and pneumonia [6, 7] have been observed during the first year following injury [3, 5, 8, 9]. Additionally, increased transitions across healthcare institutions risk continuity of care [10]. Despite significant attention to shortened lengths of acute inpatient rehabilitation (AIPR) in the late 1990s to early 2000s, recent attention to this phenomenon has waned in contemporary SCI literature.

In the quest for sufficient and appropriate care, people with SCI and their support persons face barriers accessing appropriate health and rehabilitative services in the community, ranging from limited knowledge about SCI among non-specialized practitioners to inaccessible offices, examination tables, scales, and diagnostic equipment [3, 11–14]. Inaccessible environments prevent important interventions such as weight assessment, colonoscopies, pelvic examinations, mammograms, and bone density examinations 14. Additional barriers are posed by insurance. For example, people who rely on Medicaid after SCI may have difficulty finding physicians who accept patients with Medicaid insurance [15]. These multifarious barriers, resulting in inequities in care, are social justice issues [16]. The current state of post-acute SCI rehabilitation is one in which people return to their communities with fragile health, reduced capabilities, and barriers to accessing specialized care.

### Defining sufficient and appropriate care

Consequences of paralysis have significant health and social implications for people with SCI and their families as they navigate the recovery process within a medical model-influenced healthcare system. For the purposes of this study, sufficient and appropriate care after SCI refers to addressing medical and rehabilitation needs in a comprehensive and timely manner to maximize functional recovery and minimize complications from secondary conditions. Comprehensive, in this definition, refers not only to the broad range of services required by a person with SCI, but also to the length of time that such services may be necessary, including AIPR and post-acute rehabilitation interventions.

### Health insurance and welfare in the United States

The complex U.S. health insurance and welfare systems are comprised of public and private components and can significantly influence access to sufficient and appropriate care after SCI. More than half of U.S. citizens under the age of 65 years secure health insurance privately through their employers, who often subsidize costs while requiring beneficiaries to pay a certain portion [17–19]. Private insurance often features public benefits [20]. For example, individuals who purchase health insurance through employment can benefit from federal tax policies that exclude the purchase from taxation [18, 21]. Public health insurance programs include Medicare for individuals 65 and older or younger individuals with certain disabilities, and Medicaid, a joint federal-state program for economically disadvantaged individuals. Approximately one-quarter of Americans access coverage through Medicaid or plans purchased privately, such as those made available through the Patient Protection and Affordable Care Act (PPACA) [17, 22]. Other forms of health insurance coverage include worker’s compensation benefits received in cases of work injuries, or Veterans health benefits for those with a qualifying history of military service.

Medicaid waiver programs provide home and community-based services and are distinct from Medicaid health insurance. There are more than 300 active waiver programs nationwide, offering services such as case management, personal care assistance, home modifications and equipment, and respite care [23]. Additional resources include Social Security Disability Income (SSDI) and Supplemental Security Income (SSI). SSDI provides financial benefits based on employment history, available to individuals with a disability who have accumulated a sufficient number of work credits. SSI, a safety-net program that provides a minimum income, is available to low-income individuals who have not worked or have not earned enough work credits to qualify for SSDI. Programs such as Medicaid and SSI have narrow income eligibility restrictions.

*The goal of this study is to investigate the experiences of individuals with SCI as they navigate rehabilitation, resources for recovery, and community reintegration during the first year after injury*.

## Methods

Institutional Review Board approval was obtained prior to conducting this study at a rehabilitation hospital located in the midwestern region of the United States. After informed consent, a series of in-depth semi-structured interviews were conducted over the course of one year. Participants were recruited during AIPR following a newly-acquired SCI. Eligible participants were age 18 years or older, lived within 200 miles of the rehabilitation center, and were able to communicate in English. Purposive sampling was used to recruit a diverse cohort in terms of age, sex, race, and severity of injury.

Interviews were conducted approximately every other month for one year. First interviews took place at the rehabilitation center. Subsequent interviews were conducted at a location chosen by the participant, or by phone if preferred. The initial interview queried information about the circumstances of injury and the participant’s life prior to injury, including family relationships, occupational history, and interests. Baseline information about insurance status and social benefits (i.e., Social Security Disability Income, Worker’s Compensation benefits) was obtained. Subsequent interviews omitted baseline questions. Every interview addressed the following broad topics: things that have/have not been going well, current needs, greatest concerns, greatest challenges, and expectations. At each follow-up interview, participants were asked about changes in insurance or eligibility for other resources since the previous interview. Interviews were audio-recorded and transcribed verbatim.

Data were analyzed using a constructivist grounded theory approach, which departs from traditional grounded theory by including active and equal engagement of participants in the research process and acknowledging that pre-conceived notions of the researchers influence the process [24, 25]. Interpretation was also guided by the transformative paradigm, which examines power and social structures within and across institutions and gives voice to people at risk for marginalization [26, 27]. Transcripts were reviewed multiple times to ensure familiarity with the data, and content was analyzed for emerging themes within and across participants. Analytic memo writing was undertaken to further analyze content [28]. Participants were engaged in the analytic process through informal discussions about reported phenomena, and frequently asked to provide feedback about emergent themes. This approach not only ensured accuracy of the data, but further advanced the collective voice of the study cohort.

## Results

### Demographics

Twenty participants with SCI enrolled between July 2017 and November 2019. Demographics and injury characteristics are shown in Table 1. The majority of participants are male and the median age is 43 years (range 18–64), comparable to SCI demographics in the United States [29]. The most common cause of injury for this cohort is fall, followed by vehicular accidents.

**Table 1:** Participant Demographics.

Age at Injury [mean years, range)	42.5 (19–64)
**Sex** [*n* (%)]	
Female	5 (25)
Male	15 (75)
**Race** [*n* (%)]	
Black or African American	3 (15)
Hispanic or Latino	2 (10)
White	15 (75)
**Employment Status at Injury** [*n* (%)]	
Employed	16 (80)
Unemployed	2 (10)
Retired	2 (10)
**Marital Status** [*n* (%)]	
Married	8 (40)
Committed Relationship	6 (30)
Single	6 (30)
**Education** [*n* (%)]	
Bachelor or Higher	7 (35)
Some College/Trade School	7 (35)
High School	5 (25)
Less Than High School	1 (5)
**Health Insurance Status at Injury** [*n* (%)]	
Private Insurance	14 (70)
Medicaid	4 (20)
Uninsured	2 (10)
**Injury Level at Discharge** [*n* (%)]	
Tetraplegia	16 (80)
Paraplegia	4 (20)
**AIS at Discharge** [*n* (%)]	
A	4 (20)
B	4 (20)
C	5 (25)
D	7 (35)
**Acute Inpatient Rehab LOS** (mean days, range)	46 (21–93)
**Transitions** (mean number of, range)	5.4 (2–11)
**Discharge Status** [*n* (%)]	
Home	10 (50)
Skilled Nursing Facility	10 (50)
**Residence at End of Study** [*n* (%)]	
Home	18 (90)
Skilled Nursing Facility	1 (5)
Deceased	1 (5)

*LOS* Length of stay, *AIS* American Spinal Injury Association Impairment Scale.

Emergent themes consisted of trajectories of medical and rehabilitative care, including transitions across care institutions; experiences in AIPR and discharge uncertainty; discharge to SNFs; and experiences seeking resources in the community.

### Trajectories of medical and rehabilitation management after spinal cord injury

Trajectories of post-injury medical and rehabilitation management varied across participants. Nine of 20 participants were transported to a level one trauma center immediately following injury. The remaining eleven participants were transported to a local community hospital for stabilization before transfer to a level one trauma center. Eight of the 20 participants were admitted to the acute level one trauma center affiliated with the Model SCI System within 24 h of injury and subsequently transferred to the AIPR unit. The remaining twelve were admitted to the Model System AIPR unit between four and 76 days (median 26) post-injury.

**Fig. 1:**
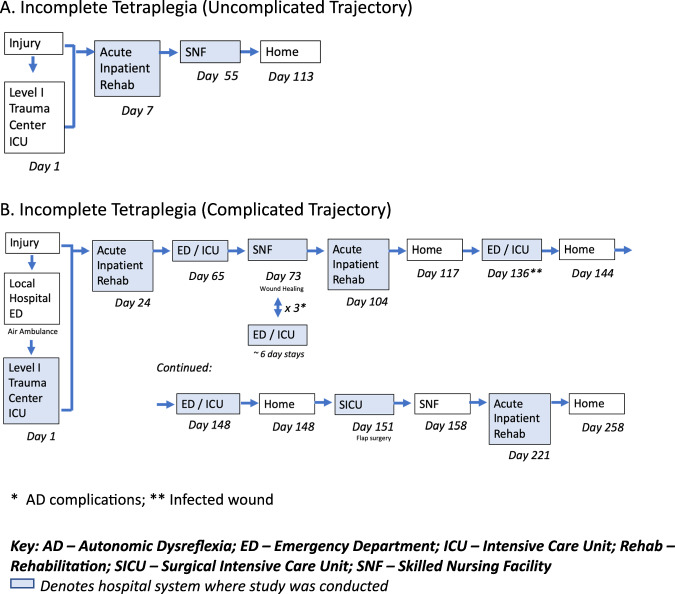
Example trajectories following spinal cord injury. **a** an uncomplicated trajectory compared to (**b**) a complicated trajectory. *AD complications; **Infected wound. AD Autonomic Dysreflexia, ED Emergency Department, ICU Intensive Care Unit, Rehab Rehabilitation, SICU Surgical Intensive Care Unit, SNF Skilled Nursing Facility. Blue shading denotes hospital system where study was conducted.

Figure 1 illustrates experiences of two participants with incomplete cervical SCI, contrasting a complicated (1B) and uncomplicated (1A) course of care. Significant to the complicated trajectory is the number of *transitions* within and across different healthcare institutions. Transitions include transfers from the site of injury to acute care, to inpatient rehabilitation, to the emergency department or medical surgical units for medical complications, or temporary transfers to SNFs or long-term acute care hospitals. Over half of the participants in this cohort experienced five or more transitions prior to discharge to home or alternative discharge setting (Table 1). The average number of transitions was five (range two – eleven).

### Transitions from acute inpatient rehabilitation

Participants and their support persons reported challenges navigating sufficient rehabilitation. The average inpatient rehabilitation length of stay (LOS) for this cohort was 46 days (range 21–93). Themes that emerged from early interviews included discharge readiness, LOS uncertainty, and insurance impacts (Table 2). Eighteen of 20 participants reported not being ready for discharge and expressed a need for additional therapy. All participants in this study experienced pressure from their insurance institution to reduce inpatient days, resulting in a peer-to-peer appeal conducted on their behalf by a physician or other rehabilitation team member to negotiate additional coverage. Some of the more severely injured participants had more than one round of negotiations for longer LOS conducted on their behalf. As shown in Table 2, participants questioned the process and accuracy around identifying a discharge date, and expressed concern about maximizing their access to rehabilitation in an environment where revenue generation plays a role and insurers challenge providers’ recommendations.

**Table 2:** Acute Inpatient Rehabilitation Experiences.

Theme: Discharge Readiness
*Everything takes time, but yet … That’s the current process (discharge). Everybody is telling me it takes time, but then you won’t provide the time for it (rehabilitation), or at least that’s how it feels to me. Participant 10, Interview 2*
*I really wanted to stay. Because I knew I was getting really good rehab. Three hours a day and … They had the music therapy too. I really did want to stay. Just those two more weeks, and then get that rehab and everything together and then go, because when I got to (skilled nursing facility). I knew this was gonna happen, I went down to one hour, even though they tell you, oh, you’ll get two hours, because this is this type of place. Participant 2, Interview 2*
When asked about readiness to leave rehabilitation: *I’ll say yes. Because physically my body I don’t think was ready for them to do anything else. Participant 12, Interview 2*
**Theme: Length of Stay Uncertainty and Insurance Impacts**
*Why do they set a discharge date when they don’t really know? You know what I’m saying? It’s just an estimate. Well, it’s all about cash revenue. Participant 12, Interview 1*
*Actually, they’re looking for an early discharge which may be even next week or sometime. I mean they are fighting. [Interviewer] And so, is it your insurance that is suggesting an early discharge? [Respondent] Not suggesting, but pressuring for it. Participant 15, Interview 1*
*Two Saturdays ago, they made a phone call here to tell us that we had been denied since the 31st of (month), and it was already like the 4th of (month). So we were like, how can you deny us from days we’ve already been here? As it is, come tomorrow they’re having to put in another report just to see if I can stay another week. Participant 14, Interview 1*
*The practical issues are that I have found it impossible how the negotiations with the insurance company went on daily. It was a daily matter about being discharged or not. So, I understood that it has become imminent and after five or ten such episodes, I said okay, I have enough of this, I’m just setting my own dismissal date and take day by day of what’s going to happen… I want to plan essentially. But then they let me know, no, no, no, I should play the game with them, and they were right, because within a week I could make the transition from not being able to sit, to sit stably, not being able to walk, to walk. So a week’s timing (affected recovery) which it wasn’t initially, so I wasn’t at that point of my recovery yet. Participant 15, Interview 2*

### Transitions to and from skilled nursing facilities

Ten of the 20 participants were discharged to a skilled nursing facility (SNF) after AIPR (Table 1). Each participant was discharged to a different SNF. All but one participant had a cervical level injury. All ten participants reported discharge to a SNF to allow more time to coordinate services for independent living, such as personal care assistance and home accessibility modifications. A close secondary factor reported was the desire for additional therapy that could be accessed at the SNF. At the time of study completion, eight participants were discharged from the SNF to a private residence, one was still in residence at a SNF, and one was deceased. Participants reported challenges during their transitions to and from SNFs. Emergent sub-themes included finding and choosing a facility, timelines associated with decision-making, and perceived premature cessation of therapy in the SNF (Table 3).

**Table 3:** Transitions to and from Skilled Nursing Facilities.

Theme: Choosing a SNF
*Okay, this place was sold as the best place to come for a spinal cord injury. Where’s the next, where’s the like step down, and what’s the best place for that step down? Where is the next place? There’s got to be a rating someplace that you guys know about, that says alright, if we’re the best, then the next best place to be is at this place. Because there’s gotta be something that’s like, I can’t be the only one that gets out of here and like can’t go home. Participant 10, Interview 1*
*My next step was supposed to be a step-down facility… you know, less than the acute rehab. [Interviewer] Like a skilled nursing facility? [Respondent] Yes, with great rehab, that’s what I was looking for. But I was trying to make choices based on ratings on the internet and I had to find a place… Participant 16, Interview 1*
*The thing is, the dead giveaway is the fact that they came to see us. Yeah. They sought out both (wife) and me, and gave us cards and stuff like that. So, they’re coming here doing their marketing. They’re in need of patients for some reason. Which they wouldn’t need to do if their, you know, rating were higher. People would be coming to see them, not vice versa. [Interviewer] Yeah. Yeah. So, how do you feel about that then? [Respondent] I feel that in combination with the fact that one of the nurses comes in and says that we need to be out of here by the end of the day today, (pause) you know, makes me feel as though I’m being railroaded. Participant 5, Interview 1*
**Theme: Timeline**
*I was in therapy, my therapy ended at 2:00 and she (social worker) says okay, you have to decide where you want to go. I said well, okay I’ll go to (SNF). She said, “Okay, your transportation is at 4:00.” Four o’clock today? “Yep, you’re leaving in two hours.” Participant 7, Interview 2*
*We were waiting on approval and we got the approval and then we had to leave late that night like. It was like okay, which we knew we were waiting on approval, but like, being have to leave that late at night, that initially bothered me, just because, night staffs are different, like … Participant 10, Interview 2*
**Theme: Early cessation of SNF services**
*The fact that they say I don’t make enough progress and therefore want to cut me from services here. So, then you either become a private payer, which is not realistic because it’s like three hundred and five dollars a day. Like they don’t think… Apparently medical necessity was thrown around, that it’s not necessarily medically necessary for me to be in a SNF. Participant 10, Interview 2*
*They didn’t give any warning. They literally walked in and it was complete jargon. I mean, absolute… I kept on asking, so why, why am I not going to be able to have any more therapy, and they said well because we feel that … She kept saying we feel that you’ve met your goals that we have, expect for you. Participant 11, Interview 2*

Choosing a SNF was a significant challenge for many participants. Commonly, participants would ask the interviewer, “What is the best SNF for spinal cord injury care?” Participants expressed a desire for quality, SCI-specific therapy in the skilled facilities as a “step-down” from AIPR. They desired guidance about appropriate facilities, and expected that health professionals in AIPR would guide them to the next level of care by providing clearly defined options for them to consider. While participants were provided with a list of facilities and star rating system from Medicare Nursing Home Compare, information about SCI-specific expertise at the facilities was not available. As a result, they or their family members needed to spend additional time researching appropriate facilities. Additionally, participants reported pressure to make rapid choices, often followed by fast transitions, with transfers to a new facility occurring within hours of the decision being made. Paradoxically, participants who sought SNF care for extended rehabilitation and therapy reported insurance coverage negotiations that mirrored their experiences in AIPR. Participants faced discharge within weeks of admission, due to insurance cessation of coverage resulting from reported lack of progress in therapy.

### Transition to outpatient services in the community

The most commonly reported experiences in the community were related to transitions to outpatient therapy services. Out of ten participants who were discharged directly home from AIPR, seven accessed outpatient therapy at the SCI rehabilitation center where they completed AIPR. Facilitators included personal financial resources to purchase an accessible vehicle (1 participant), accessible transportation services paid for by workers’ compensation (1 participant), functional ability to transfer into conventional vehicle (4 participants), or financial resources to stay in an accessible apartment adjacent to the rehabilitation facility, negating need for transportation (1 participant). Out of the three participants who did not access outpatient therapy at the SCI rehabilitation center, one was restricted by insurance to an outpatient facility at another healthcare institution (and had resources to purchase an accessible vehicle); one lived an hour from the rehabilitation facility, and while able to transfer into a car, opted for a closer therapy location to reduce transportation costs and strain on family; and one had private insurance that did not pay for transportation, lacked access to a modified van, and was unable to be transferred into a car. This individual received occupational and physical therapy services in the home.

As indicated previously, eight of the ten participants who were admitted to a SNF after AIPR ultimately left the facility to live in the community. After discharge from the SNF, these participants sought additional therapy in the community. Seven of these eight individuals eventually accessed outpatient therapy at the SCI rehabilitation facility where they received inpatient rehabilitation, five of whom received home therapy services first, while managing logistics for transportation. One participant received home therapy exclusively due to persistent transportation barriers. Despite having access to wheelchair transportation services through Medicaid’s Home and Community-based Services Waiver Program, he was unable to find a company that would serve his geographic region.

Finally, participants expressed significant resource navigation challenges, including accessing information and managing many concerns at one time highlighting additional work and emotional stress concurrent with efforts to recover (Table 4).

**Table 4:** Resource navigation challenges.

Theme: Communication and Accessing Information
*So, you’re always going through somebody to talk to somebody else, and you never really feel like you’re getting the whole story, and that’s what it felt like. I thought today like, okay, well if I can’t get in touch with my case manager, just call member services, that’s like the hub of case managers, right, you know. But no, you have to talk to your case manager. Well, you can’t ever get one of them on the phone right when you first call them. It’s always call, leave a message… Wait until they call back. Participant 11, Interview 3*
*I’d call, you never could get through to anybody. And then you’d leave a message and then it would be two days later and somebody would call you back. Well, if I couldn’t get to the phone, if I was at therapy, if I was, just couldn’t get to the phone in time, then I had to call back, and it was another two days. They were ridiculous. They must be obviously busy and understaffed. Participant 1, Interview 3*
*[Interviewer] How often do you think you’re on the phone like making calls for things, would you say? [Respondent] Um, usually we do it after lunch. Some days we’ll spend like more time than other days, write a list of people we need to call. There’s a lot of waiting on hold, leaving voicemails. Participant 11, Interview 2*
*Well, I’m gonna be getting on the ball here, if not today, tomorrow. I have to get this going and I just, I’m just pissed because they don’t call back. Participant 6, Interview 1*
*It’s a circle, it’s just like circles inside of circles inside of circles. Like you get one number to call and they can’t help you, so they give you another number. I got a phone number. I called that phone number. The guy would not like, he would not give me any information. He just said I had to be transferred to this different person. So, then he transfers me to the different person. Of course, the different person doesn’t pick up, so then I had to play phone tag with her for like a day. I had reached my level of call insanity. Participant 10, Interview 6*
*It’s like, these are the things I need right now to be functional in the future. Like if I want to get to a point where I can work, if I want to get to a point where I can have an independent life, like I need these things. You know all these… everything, yeah, it just seems like it’s hard. It’s hard to get there. Participant 2, Interview 3*
**Theme: Navigating Many Concerns at Once**
*Oh, there’s a list already. Um, what happens next? Ummm so my mom does have a handicap accessible house in (city), but whether that’s the best option, or whether, or what are the options…. So like, where do I actually go next? If I end up at like a facility, where is the best facility, uhhh because I know. They’re hit or miss and like it’s very variable. Like some are really good, some are meh, and then there are some that are just kind of awful. What happens with work? Like if I wanted to be more indep - like if I didn’t want to rely on my mom, is there an independent option? Participant 10, Interview 1*
*There’s a million challenges right now … Participant 12, Interview 2*
*It honestly feels like at this point we’re just trying to figure out one issue as it comes and kind of manage getting through the days and weeks and so like, future thinking really isn’t happening as much as it probably could. Participant 2, Interview 3*
*Don’t know what is real, what is possible, what is effective. But decisions need to be made. We prefer to make informed and good decisions. Participant 15, Interview 1*
*I haven’t even thought about it. Yeah. I can only climb one mountain at a time. Participant 16, Interview 1. I can’t just dive into everything at the same time. Participant 16, Interview 2*
*Like I said, my only goal right now, I’ll deal with everything else after the fact, is to get home to my wife and kids and become the man that I was before. Health wise. That’s what I said, I just take it one step at a time now, and get home and then I’ll deal with that. Participant 17, Interview 1*
*Just, it’s hard enough to just … To (focus on today). Just between the pain, exhaustion, and damn insurance companies, and then the housing. Participant 11, Interview 1*

## Discussion

This study illuminates multifactorial challenges experienced by people with SCI during the first year following injury, from their own perspectives, demonstrating an important application of the transformative paradigm. Medical and physical impacts of SCI are well documented in the literature, but there is little information about navigational experiences or activities undertaken while seeking recovery, including transitions across institutions, the hidden work expended in order to navigate health and social resources [30], and the emotional toll of persistently experienced barriers to societal participation. This study highlights challenges experienced by people with SCI across transitions in care that risk access to sufficient and appropriate care, revealing important imbalances in power between these individuals and institutions involved in their rehabilitation.

### Post-injury trajectories and significance of transitions

In many cases, post-injury trajectories were complicated and included numerous transitions in care. Participants with more complex injuries or secondary conditions experienced a greater number of transitions within and across health care systems, risking discontinuity of care [10]. Such transitions can have adverse impacts on safety and wellbeing through increased readmissions, medication mistakes, and greater health care spending [10, 31–33]. Studies have shown that individuals with complex conditions and their caregivers often feel unprepared to manage their condition after hospitalization, due to a paucity of information and reduced access to health professionals to direct questions [31]. This is a salient issue for individuals with SCI, who experience barriers accessing knowledgeable practitioners or SCI-specialized care outside of their rehabilitation facility [14].

Importantly, while all participants were recruited from a SCI Model Systems Center for rehabilitation [34], they had different trajectories immediately following the injury. Slightly less than half of the cohort were taken immediately to a level one trauma center that would offer the best level of care for acute SCI. The rest were transported to local community hospitals before accessing a higher level care. Additionally, despite access to SCI-specialized treatment during inpatient rehabilitation, this cohort still experienced barriers to sufficient and appropriate care, causing great concern for individuals with SCI with limited or no access to specialized SCI care within their communities.

### Transitions from acute inpatient rehabilitation: who is making decisions about care?

Results for this cohort show slightly longer AIPR lengths of stay (average of three days) compared to typical SCI experiences [29], likely due to the high number of participants with tetraplegia. However, the current trend of decreasing lengths of stay for AIPR was reflected in the experiences of this cohort, including LOS uncertainty, perceived discharge unreadiness, and influence of insurance institutions. The majority of participants expressed a desire for additional rehabilitation, questioning the timing of their discharge as well as how they would receive additional rehabilitation following discharge. Unfortunately, barriers to sufficient and appropriate rehabilitation arise in part from the complex relationship between eligibility requirements for AIPR services, insurance institutions, and the U.S. healthcare system, which is market-based and therefore influenced by political-economic factors [20]. Eligibility for AIPR is contingent upon the need for medical management [35]. When medical management is no longer a necessity, third party payment ceases, regardless of proficiency in skills that promote living independently. This phenomenon significantly influenced lengths of stay and access to rehabilitation, not only in AIPR, but also in SNF settings. Individuals with cervical level injury were most negatively affected, most of whom reported not being ready to leave rehabilitation, or felt their rehabilitation to be incomplete at discharge. The nature of how individuals with SCI qualify for AIPR is at odds with their longer-term needs for rehabilitation once medical and nursing needs have been met. This negates the value of therapy for functional skills development to increase proficiency in mobility, transfers, activities of daily living, and successful community reintegration. Once discharged, many individuals encounter significant barriers to SCI-specialized interventions in their community, losing the opportunity to maximize independence. While most of the participants in this study ultimately gained access to outpatient therapy at the SCI Model center, the larger population of people with SCI do not have access to these centers.

An interesting finding of this study is the high prevalence of peer-to-peer negotiations between the treating physician and a physician representative of the insurance institution who is rarely specialized in SCI, or even Physical Medicine and Rehabilitation [36]. Participants and their care teams lacked control over the rehabilitation timeline, in part, due to institutional practices of non-specialized physicians subverting expert opinions of treating physicians. This reflects a surprisingly unquestioned conflict of interest. Further, such denials of sufficient and appropriate care persist regardless of participants’ adherence to paying health insurance premiums and co-payments for services, raising the question of rights to care. The trend of decreasing lengths of stay over the past 40 years has led to a new generation of medical and allied health providers that have been raised in this constrained model of rehabilitation provision without historical experience of longer lengths of stay. Today, providers estimate discharge dates in part based on their experience regarding what insurers will pay, yet still find themselves advocating for their patients in time-consuming peer to peer meetings with insurance staff.

### Transitions to and from skilled nursing facilities

Despite ten participants being discharged after AIPR to ten *different* SNFs, participants universally reported difficult experiences. Participants and their families undertook high levels of beneficiary work [30], the often invisible and unremunerated effort that is expended to identify resources and navigate institutions in order to identify an appropriate facility. Even with tools such as Medicare Nursing Home Compare [37], participants struggled to identify whether facilities had experience caring for people with SCI. Additionally, the volume of facilities can be overwhelming to investigate, especially when “fast” decisions are needed, placing time constraints on the ability to perform necessary research into various facilities.

In association with reduced lengths of stay in AIPR, participants chose admission to a SNF for increased time to prepare for independent living in the community, and secondarily, to access additional therapy. While the amount of occupational and physical therapy in SNFs is less than what is provided in AIPR, participants anticipated it to be greater than what would be accessed via outpatient therapy. Several participants experienced early discharge, which may be associated with a lack of SCI specialization in SNF staff. Absence of specialized knowledge can manifest in a reduced knowledge about expected functional outcomes, and inadequate goal identification to justify appropriate treatment to insurance institutions. Based on experiences of participants in this cohort, SNFs are largely ill-equipped to provide sufficient or appropriate care for the complex condition of SCI.

### Limitations

This study reveals experiences of participants, most of who have cervical SCI, from one regional SCI rehabilitation center in the Midwestern U.S. Data may not reflect experiences in other geographic regions, or of individuals with lower-level SCI.

## Conclusion

A paradigmatic change is needed to promote sufficient and appropriate care for people with SCI. Trajectories following injury feature transitions across care systems that can be fraught with stress and risk discontinuity of care. The experiences of this cohort reveal significant challenges attaining sufficient and appropriate rehabilitation. AIPR is a critical aspect of recovery, but does not ensure sufficient intervention for maximization of functional skills and community reintegration. Given that this cohort was recruited from a SCI model system of care, situations for accessing SCI-specialized care are arguably worse for individuals outside of SCI model systems, which make up the majority of individuals living with SCI. There is much work to be done to maximize rehabilitation access by people with SCI. Decision-making about care should be placed in the hands of SCI providers and their patients, challenging conflicts of interest from physician representatives of insurance institutions. Additional research is needed to investigate innovative models of rehabilitation provision to people with SCI to make positive impacts on recovery trajectories and successful transition to independent living in the community.

## Data Availability

The in-depth qualitative data generated from this study are not publicly available due to concern of identifying participants. Data can be made available by the corresponding author upon reasonable request.
